# Patient safety: knowledge between multiprofessional residents

**DOI:** 10.1590/S1679-45082017AO3871

**Published:** 2017

**Authors:** João Lucas Campos de Oliveira, Simone Viana da Silva, Pamela Regina dos Santos, Laura Misue Matsuda, Nelsi Salete Tonini, Anair Lazzari Nicola

**Affiliations:** 1 Universidade Estadual do Oeste do Paraná, Cascavel, PR, Brazil.; 2 Universidade Estadual de Maringá, Maringá, PR, Brazil.

**Keywords:** Patient safety, Internship and residency, Knowledge management

## Abstract

**Objective:**

To assess the knowledge of multiprofesional residents in health about the security of the patient theme.

**Methods:**

Cross-sectional study, quantitative, developed with graduate courses/residence specialties of health in a public university of Paraná, Brazil. Participants (n=78) answered a questionnaire containing nine objective questions related to patient safety. Data were analyzed using descriptive statistics, in proportion measures. The minimum 75% of correct answers was considered the cutoff for positive evaluation.

**Results:**

The sample was predominantly composed of young people from medical programs. Almost half of the items evaluated (n=5) achieved the established positive pattern, especially those who dealt with the hand hygiene moments (98.8%) and goal of the Patient Safety National Program (92.3%). The identification of the patient was the worst rated item (37.7%). In the analysis by professional areas, only the Nursing reached the standard of hits established.

**Conclusion:**

Knowledge of the residents was threshold.

## RESUMO

**Objetivo:**

Verificar o conhecimento de residentes multiprofissionais na área da saúde sobre o tema segurança do paciente.

**Métodos:**

Estudo transversal, quantitativo, desenvolvido com pós-graduandos dos cursos/especialidades de residência da área da saúde de uma universidade pública do Paraná. Os participantes (n=78) responderam um questionário contendo nove questões objetivas relacionadas com a segurança do paciente. Os dados foram analisados por estatística descritiva, em medidas de proporção. O mínimo de 75% de acertos foi considerado ponto de corte para avaliação positiva.

**Resultados:**

A amostra foi composta por profissionais predominantemente jovens, oriundos de programas médicos. Quase metade dos itens avaliados (n=5) alcançou o padrão de positividade estabelecido, com destaque para os que trataram dos momentos de higienização das mãos (98,8%) e o objetivo do Programa Nacional de Segurança do Paciente (92,3%). A identificação do paciente foi o pior item avaliado (37,7%). Na análise por áreas profissionais, somente a Enfermagem alcançou o padrão de acertos estabelecido.

**Conclusão:**

O conhecimento dos residentes foi limiar.

## INTRODUCTION

Patient safety is currently considered one of the critical pillars of quality in health care. It is undeniable that health care services can be potentially harmful, and patient safety is defined as reducing health care-associated risks to an acceptable minimum.^1^

To improve safety in patient care, some strategies are recommended aiming at standardizing work processes; identifying risks and planning services; critically reviewing scientific content; building commitment towards this purpose, including a non-punishing culture concerning mistakes; and a better communication between workers and users.^1-4^

Health professionals must have the knowledge and skills to identify mistakes and take appropriate action to reverse them and prevent them in time, promoting changes in the safety culture of organizations.^3,5^ Recently, training in patient safety has become mandatory in undergraduate and graduate health-related syllabus in Brazil.^1^

The importance of diagnosing/assessing the level of patient safety knowledge of healthcare professionals is well recognized. However, in the context of both education and research, this has been addressed only for undergraduate students - particularly in Medicine and Nursing.^5-7^ Bohomol et al., *e.g.*, performed a similar analysis for additional areas of expertise (Pharmacy and Physiotherapy) other than those already mentioned, focusing on undergraduate students.^8^

This study focuses on patient safety knowledge among multiprofessional health care residents and is justified by the fact that the detection of faults and strengths in the training of these agents can possibly support the decision-making process when looking for ways to improve patient safety. The subjects of this study are still in school, even if on practical training, and can become agents of change in their fields in the future. With this in mind, we asked the following question: what is the level of knowledge (or lack of) of patient safety among multiprofessional health care residents?

## OBJECTIVE

To check the knowledge of multi-professional residents on the topic of patient safety.

## METHODS

A descriptive, cross-sectional, quantitative study based on health care residency programs offered in a university in the countryside of the state of Paraná. Among the graduate students in these programs, except for the field of industrial pharmacy, all of them were undergoing in-service training (which is a characteristic of residency programs) in a university-hospital.

The hospital has 210 beds for medium and high complexity services exclusively under Brazil’s public health system (SUS - *Sistema Único de Saúde*). It receives 51 students every year for residency programs in five areas: Medicine, Nursing, Physiotherapy, Pharmacy and Dentistry. Under these areas, residency programs are subdivided into 14 specialties related to Medicine (n=7), Nursing (n=1), Physiotherapy (n=2), Pharmacy (n=3) and Dentistry (n=1). At the time of the study, there were 113 (100%) active residents.

The study population comprised all the residents who were active during data collection, which took place from March to May 2016. Subject enrollment was based on the active registration of residents and their attendance of theoretical and/or practical classes. On that basis, we excluded any residents who were absent from campus due to vacations/leaves of absence; who failed to answer the questionnaire (data collection) after three consecutive invitations; who could not be found at their in-service training sites or the classroom after three attempts.

To reach the subjects, we formally requested a list of names to the coordinator of each area of expertise/specialty. Potential respondents were recruited at their in-service training site and/or classrooms, and invited to participate in the study after learning its objectives and methods. After informally accepting to take part, they were handed the Informed Consent Form to read and sign in two copies, which were also signed by the investigator.

The data collection was conducted with a self-administered questionnaire including collection of demographic data and details on the respondent’s educational background. There was also a specific session about patient safety, based on official national standards available on this topic, with nine multiple-choice questions of four alternatives (a-b-c-d), and only one correct answer (Appendix A).^1,9,10^

The specific questions (n=9) of the questionnaire covered the following evaluation points: purpose of the National Program for Patient Safety (PNSP - *Programa Nacional de Segurança do Paciente*); risk of infection; major patient safety goals/protocols established by the National Health Surveillance Agency (ANVISA - *Agência Nacional de Vigilância Sanitária*); times when hand washing is required; times when the safe surgery checklist is required; patient safety taxonomy (focus on mistakes and adverse events); pressure ulcers (grading stages); recommendations for patient identification; and fall prevention.^1,9,10^

To verify its suitability, the questionnaire was previously applied to a random sample of six subjects in a pilot test among undergraduate health students, which corroborated the usability of the questionnaire. In addition, the questionnaire was evaluated by three nursing professors: two PhDs and one doctorate student with a Master’s degree. They unanimously approved the questionnaire for research purposes. In the future, we expect the questionnaire to be subjected to and fine-tuned by appropriate validation procedures, which is certainly recommended, since there are no other questionnaires available for the same purpose.

The data extracted from the questionnaires were summarized in Microsoft Excel 2010 spreadsheets and subsequently submitted to a descriptive statistical analysis with proportion calculation. To ensure objectivity, we established 75% as a cut off percentage for a positive rating of the respondents’ knowledge. We chose this number because we were not aware of any guidelines establishing evaluation parameters for patient safety knowledge, and also because it is the same cut off used by another questionnaire about the safety climate for hospitalized patients, a topic which is somehow related with the theme of this study.^11^

All ethical requirements established by Resolution 466/2012 of the National Health Council were met and the study design was appraised by the Ethics Committee of *Universidade Estadual do Oeste do Paraná*, and approved under number 1311148/2015, CAAE: 48192015.9.0000.0107.

## RESULTS

By eligibility, the study included a sample of 78 respondents or 69% of the pre-eligible population (n=113). Sample losses were due to the following reasons: residents on vacation/leaves of absence (n=11); not found at the in-service training site and/or classroom after three attempts (n=6); and those who failed to answer the questionnaire after three invitations (n=18).

Table 1 contains information about the respondents such as age, gender, years of residency and major. Table 2 shows the distribution of respondents according to the residency specialties studied. Table 3 contains information on the residents’ knowledge of patient safety, by item evaluated. Finally, table 4 summarizes their knowledge of patient safety by training area, *i.e*., by the individual frequency of answers in each area.

**Table 1:** Sample characterization

Variable	n (%)
Age	
20-23	24 (30.7)
24-27	41 (52.5)
28-31	9 (11.5)
32-35	3 (3.8)
Did not answer	1 (1.5)
Gender	
Female	53 (67.9)
Male	25 (32.1)
Years of residency training	
R1	53 (67.9)
R2	22 (28.3)
R3	3 (3.8)
Major	
Medicine	29 (37.2)
Nursing	13 (16.7)
Physiotherapy	15 (19.2)
Pharmacy	15 (19.2)
Dentistry	6 (7.7)

Total	78 (100)

**Table 2:** Respondents’ specialties

Residency specialty	n (%)
General Surgery	4 (5.1)
Internal Medicine	3 (3.9)
Neurosurgery	2 (2.5)
Obstetrics and Gynecology	11 (14.1)
Orthopedics and Traumatology	3 (3.9)
Pediatrics	6 (7.6)
Medical/Surgical Nursing Management	13 (16.6)
Inpatient Physiotherapy	10 (12.8)
Physiotherapy in intensive care	5 (6.5)
Clinical Analysis.	3 (3.9)
Hospital Pharmacy	9 (11.6)
Oral and Maxillofacial Surgery and Traumatology	6 (7.6)
Industrial Pharmacy	3 (3.9)

Total	78 (100)

**Table 3:** Knowledge of patient safety among multiprofessional residents

Subject of the question	Correct answers n (%)	Incorrect answers n (%)	Total n (%)
Purpose of the PNSP	72 (92.3)	6 (7.7)	78 (100)
Risk of infection	63 (80.8)	15 (19.2)	78 (100)
Primary patient safety goals (ANVISA)	66 (84.7)	12 (15.3)	78 (100)
Times when hand washing is required	77 (98.8)	1 (1.2)	78 (100)
Safe surgery checklist	55 (70.5)	23 (29.5)	78 (100)
Patient safety taxonomy (mistakes and adverse events)	47 (60.2)	31 (39.8)	78 (100)
Pressure ulcers	60 (77)	18 (23)	78 (100)
Patient identification	29 (37.7)	48 (62.3)	77 (100)*
Fall prevention	57 (74)	20 (26)	77 (100)*

* Blank items were not considered.

PNSP: *Programa Nacional de Segurança do Paciente*; ANVISA: *Agência Nacional de Vigilância Sanitária*.

**Table 4:** Knowledge about patient safety among multiprofessional residents, by professional area

Major	Correct answers n (%)	Incorrect answers n (%)	Total n (%)
Medicine	191 (73.8)	68 (26.2)	259 (100)
Nursing	105 (89.8)	12 (10.2)	117 (100)
Physiotherapy	100 (74)	35 (26)	135 (100)
Pharmacy	96 (71)	39 (29)	135 (100)
Dentistry	34 (63)	20 (37)	54 (100)

## DISCUSSION

Most of the respondents in the sample were young. One of the possible reasons is that residency programs are originally developed for newly graduated students, who are usually young. This finding is also explained by the fact that most respondents were in their first year of residency. Although isolated, this data suggests that most of the respondents had graduated quite recently and, in theory, should have recently studied content related with patient safety.

Patient safety training has been mandatory in Brazil since 2013, *i.e.*, a period which includes the graduation of most of the respondents - considering their ages.^1,9^ It may be that some higher education institutions are still undergoing revisions of the curricula and contents which is very important in the context of patient safety, since this topic goes beyond the technical lessons that are still quite present in health-related education programs. According to the national recommendation, residents must have the opportunity to learn contents related to patient safety practices and management also during residency training, as part of their post graduate education in healthcare.^1^

It is noteworthy that the largest concentration of respondents were medical residents, which is certainly linked to the largest number of specialties offered in this professional field. Actually, residency training, *i.e.* in-service training in health care as a form of specialization originated from this discipline. ^12^

As for patient safety knowledge, the first item evaluated by respondents was about the Ordinance of the Ministry of Health establishing the PNSP. One of its main objectives as to promote a support the implementation of initiatives related to patients´ safety in different care areas, organization and management of healthcare services.^1^ high percentage of correct answers was observed for this item, revealing the widespread dissemination of general information or the easy identification of the basic purpose of the PNSP. This piece of data, however, cannot be considered as knowledge that legitimizes best health practices, although it attests to the successful publicity of an important policy governing such practices, and it certainly deserves to be disseminated as much as possible among health providers, including residents.

The worst rated item of the residents’ evaluation was the recommendations for patient identification. This was a particular concern, since said process is critical for provision of planned care to the right patient, but potential medical errors cannot be minimized if the health care staff fails to rationally and critically apply the right form of identification, which is currently through bracelets.^10,13^ Thus, the fact that more than half of the residents do not know how to properly identify patients is alarming because it points to the need to priorly check whether the identification method chosen is correct, which could result in poor adherence to this safety barrier.

In agreement with the aforementioned, a study carried out at the same location of the present study showed that, of 1,068 inpatients investigated, 250 (or about 24% of the sample) did not have any of the predefined identifiers (on the bed or bracelet), meaning that those patients were certainly more exposed to mistakes and adverse events.^14^ This implies that the low level of knowledge of residents is alarming since, otherwise, they could be agents working to improve adherence at their organizations.

A recent study conducted at a large hospital in the state of Rio Grande do Sul with the purpose of determining the impact of educational initiatives on the indicator for adherence to verification of patients’ identification bracelets before any high-risk procedure detected major improvements in the proposed intervention, with increased adherence over time for up to 94.37% of the sample. This led to the conclusion that these strategies based on awareness-raising among employees can potentially improve compliance with this practice and consequently lead to better patient safety.^15^ Although the result of the study does not reflect adherence directly, but rather the subjects’ knowledge of the identification procedure, this may be a good reason to train residents and possibly expand this proposal to the entire hospital.

The positive results in this study for the item related with times when hand washing is required reinforce the positive rating of the item ‘risk of infection’. This is commendable because hand washing itself is recognized as one of the most cost-effective safety barriers to prevent infections; however, just knowing how important this procedure is to improve patient safety does not ensure adherence.^10,16^ The results of this study are limited to describing the residents’ theoretical knowledge, and it is not possible to know how much this applies to their practice in terms of adherence to hand washing when required, as well as other safety actions, which can even be described as a limitation of this investigation.

The item ‘safe surgery checklist’ did not reach the cut off for a positive evaluation. This may be linked to the fact that this stage of the safe surgery protocol corresponds to the perioperative period which is often accompanied only by medical and nursing professionals.^10^ It may be that respondents from other majors did not have enough knowledge of how to use this tool.

In addition to physicians, nurses and the surgical team, dentists must also be attentive to surgical safety, since interventions of this type are also part of their scope. Since the focal knowledge of each professional class about isolated items was not measured, it is impossible to say that dentistry residents were unaware of the safe surgery checklist, even if the evaluation by professional area was negative, which could be an interesting subject for future studies.

For the item patient safety taxonomy, which focused on the definition of mistakes and adverse events, the results were superior to those of a study conducted in the countryside of the state of São Paulo with undergraduate nursing students, who were more frequently unaware of the term ‘adverse events’ when compared with the term ‘mistakes’.^6^ Residency training is, therefore, an important means to educate people in their practice and, added to the scientific knowledge offered in specialization programs, it can add value to an organization’s human capital, improving people’s ability to reflect, think critically and solve problems.

Nursing residents had the highest level of knowledge of patient safety. The reason for this finding may be linked to the fact that nurses are those who spend the most time in direct contact with users; are involved in all care processes; and have management functions including rigorous quality management activities and the implementation of cyclic and systematic strategies to ensure patient safety.^2,17^

Generally speaking, the professionals (Nurses, Physicians and Physical Therapists) who are frequently in direct contact with hospitalized patients had the best level of knowledge of safe patient care, which may explain the good results found for the item related with pressure ulcers, and also fall prevention which, differently from patient identification (with a low rate of correct answers), are safety strategies used in a much more clinical/applied setting. This is relevant because during residency training these professionals are still in school and this could promote the incorporation of a safety-friendly culture through the development of clinical/patient care skills supported by scientific knowledge.^3^

We recommend that the programs investigated, particularly Dentistry and Pharmacy, revisit their teaching-learning plans focusing on patient safety, considering that these professionals will also impact the quality of patient care as well as patient safety. Because we cannot generalize the findings of this study, we suggest that analytical studies be conducted focused on the knowledge students/residents/professionals have of direct results on service quality and patient safety.

## CONCLUSION

Knowledge of patient safety among multiprofessional residents was borderline satisfactory, since almost half of the items reached the minimum cut off for a positive evaluation, and nursing was the only training area that reached the standard established for this study. Therefore, even while still in school, nurses were considered the most skilled professionals to perform strategic management actions aiming at safer healthcare services.

## Appendix A

**Data Collection Form**

**Table:** Data Collection Form

Instructions for completing the questionnaire			
This questionnaire will assess your knowledge of patient safety. Please sign the informed consent form, fill out some details of your residency training and then answer a few questions about patient safety. Completing this questionnaire should take between 10 and 15 minutes.			
**Patient safety** means reducing health care-associated risks to an acceptable minimum.			
**1) Age:** ____years old.			
**2) Gender:** [ ] Female [ ] Male			
3) Years of residency:			
[ ] R1 [ ] R2 [ ]R3 [ ]R4 [ ] R5			
4) What is your residency program?			
a) Medicine [ ]			
b) Nursing [ ]			
c) Physical therapy [ ]			
d) Pharmacy [ ]			
e) Dentistry [ ]			
**5) What is your residency specialty?**			
a) General Surgery [ ]			
b) Internal Medicine [ ]			
c) Neurosurgery [ ]			
d) Obstetrics and Gynecology [ ]			
e) Orthopedics and Traumatology [ ]			
f) Pediatrics [ ]			
g) Cardiology [ ]			
h) Medical/Surgical Nursing Management [ ]			
i) Hospital Physiotherapy [ ]			
j) Physiotherapy in intensive care [ ]			
k) Clinical Analysis [ ]			
l) Hospital Pharmacy [ ]			
m) Oral and Maxillofacial Surgery and Traumatology [ ]			
**For every question there is only one possible answer. Thank you!**			
**6) On April 1, 2013, the Ministry of Health created the National Program for Patient Safety (PNSP) through Ordinance 529. The purpose of the PNSP is:**			
a) To promote and support the implementation of patient safety initiatives in different areas of provision, organization and management of health care services.			
b) To promote the inclusion of patient safety as a topic in higher education in health care.			
c) Make patients take on responsibility for their safety as primary providers.			
d) Build commitment and give priority to reducing infections.			
**7) One of the main points of the National Program for Patient Safety is hand washing as a means to prevent infections. Its focus is:**			
a) Explaining that the power to reduce health care-associated infections is in the hands of patients only.			
b) Increasing awareness of the impact of health care-associated infections in order to reduce their incidence.			
c) Minimizing the importance of other sources of infections, since the primary cause is poor or no hand washing.			
d) Strictly following protocols in order to reduce the number of health care-associated infections reported.			
**8) What are the key patient safety strategies set forth by ANVISA?**			
a) Hand washing, patient identification, effective communication; saving lives.			
b) Fall prevention, pressure ulcer prevention, safe drug administration, safe use of intravenous devices, ICC.			
c) Safe surgical procedures, safe administration of blood and blood products, safe use of equipment, patient monitoring and hygiene.			
d) Correct patient identification, improving communication skills of health care professionals, hand washing, safe drug administration, safe surgical procedures, reducing the risk of falls and pressure ulcers.			
**9) Hand washing is one of the most important practices in health care services. The ANVISA and the World Health Organization have defined when this procedure is required, as follows:**			
a) Before any contact with the patient, before performing aseptic procedures, after potential exposure to body fluids, after any contact with the patient, and after any contact with areas close to the patient.			
b) Before any contact with the patient, before performing septic procedures, and after potential exposure to body fluids.			
c) Before any contact with the patient if needed, in case of procedures requiring contact with body fluids, and before performing aseptic procedures.			
d) Before any contact with isolated patients, after any contact with the patient; if wearing gloves, there is no need for hand washing.			
**10) The use of checklists in surgical procedures has numerous advantages, helping the health care staff reduce the possibility of patient harm during postoperative care. The safe surgery checklist is:**			
a) A single checklist that cannot be adapted to particularities of the service and is used at three different times: before induction of anesthesia, before the surgical incision, and before the patient leaves the operating suite.			
b) A single checklist that can be adapted to particularities of the service and is mostly applied at two different times: before induction of anesthesia and before the patient leaves the operating suite.			
c) A single checklist that can be adapted to particularities of the service and is used at three different times: before induction of anesthesia, before the surgical incision, and before the patient leaves the operating suite.			
d) A single checklist that cannot be adapted to particularities of the service and is used at four different times: before surgical induction, before the skin incision, before the patient leaves the operating suite, and after the patient is transferred back to the apartment.			
**11) Patient safety has become a worldwide movement, demanding the establishment of a common language to support effective communication in health care facilities. Choose the correct definition:**			
a) An incident without injury is an event that did not affect the patient and caused no discernible damage.			
b) An adverse event is an event or circumstance that occurs sporadically, without directly affecting the patient.			
c) A near miss is an incident that affected the patient.			
d) A mistake is defined as a failure to execute an action plan as intended, or implementation of the wrong plan.			
**12) A pressure ulcer is any lesion on the skin and/or underlying tissues, usually developing over a bony prominence, as a result of pressure alone, or pressure in combination with friction and shear. Thus, one can say that:**			
a) It has four stages, namely: (I) intact skin with non-blanchable erythema, (II) partial loss of skin thickness, (iii) total loss of skin thickness, (iv) complete loss of tissue thickness with exposed bones, tendons and/or muscles.			
b) The wound can progress until it is covered by a thin layer of necrotic tissue (eschar). Its evolution is slow without exposing other layers of tissue.			
c) The depth of a stage IV pressure ulcer does not necessarily vary at different anatomical sites. They are often cavitated and fistulized.			
d) There is no scale to assess the risk of developing pressure ulcers, the only recommendation is a daily physical examination of the patient.			
**13) The Patient Identification Protocol defined by ANVISA recommends for this safety barrier:**			
a) At least one identifier printed on a white or other color bracelet according to the institution’s standard.			
b) At least two identifiers on a standardized white bracelet placed on one of the patient’s limb for checking before any procedure.			
c) The bed and chart number are the recommended identifiers to be printed on the patient’s bracelet.			
d) Colored alert bracelets or tags may be used to identify the patient, because their good visibility helps reduce the risk of misidentification.			
**For every question there is only one possible answer. Thank you!**			
**14) The purpose of the Fall Prevention Protocol is to reduce the risk and consequences of patients falling at health care facilities. On this, one can say that:**			
a) Fall risk is assessed only at patient admission using a scale deemed appropriate for the profile of the institution’s patients.			
b) The fall risk assessment scales are universal to all patient groups, *e.g*, adult and pediatric.			
c) The most commonly used scale is the Morse scale. It assesses the factors leading to falls, allowing for the rating of a patient’s risk of falling and implementation of measures required to eliminate this risk.			
d) The health care facility is not responsible for providing resources for fall prevention, which is the exclusive responsibility of the staff.			
**”ANSWERS” TO PATIENT SAFETY QUESTIONS**			
	**Question**	**Correct answer**	
	6	A	
	7	B	
	8	D	
	9	A	
	10	C	
	11	D	
	12	A	
	13	B	
	14	C	
